# Maysin and Its Flavonoid Derivative from *Centipedegrass* Attenuates Amyloid Plaques by Inducting Humoral Immune Response with Th2 Skewed Cytokine Response in the Tg (APPswe, PS1dE9) Alzheimer’s Mouse Model

**DOI:** 10.1371/journal.pone.0169509

**Published:** 2017-01-10

**Authors:** Yuno Song, Hong-Duck Kim, Min-Kwon Lee, Il-Hwa Hong, Chung-Kil Won, Hyoung-Woo Bai, Seung Sik Lee, SungBeom Lee, Byung Yeoup Chung, Jae-Hyeon Cho

**Affiliations:** 1 Institute of Animal Medicine, College of Veterinary Medicine, Gyeongsang National University, Jinju, Korea; 2 Department of Environmental Health Science, New York Medical College, Valhalla, New York, United States of America; 3 Advanced Radiation Technology Institute, Korea Atomic Energy Institute, Jeongeup, Korea; Cedars-Sinai Medical Center, Maxine-Dunitz Neurosurgical Institute, UNITED STATES

## Abstract

Alzheimer’s disease (AD) is a slow, progressive neurodegenerative disease and the most common type of dementia in the elderly. The etiology of AD and its underlying mechanism are still not clear. In a previous study, we found that an ethyl acetate extract of *Centipedegrass* (CG) (i.e., EA-CG) contained 4 types of Maysin derivatives, including Luteolin, Isoorientin, Rhamnosylisoorientin, and Derhamnosylmaysin, and showed protective effects against Amyloid beta (Aβ) by inhibiting oligomeric Aβ in cellular and in vitro models. Here, we examined the preventative effects of EA-CG treatment on the Aβ burden in the Tg (Mo/Hu APPswe PS1dE9) AD mouse model. We have investigated the EA-CG efficacy as novel anti-AD likely preventing amyloid plaques using immunofluorescence staining to visually analyze Aβ40/42 and fibril formation with Thioflavin-S or 6E10 which are the profile of immunoreactivity against epitope Aβ1–16 or neuritic plaque, the quantitation of humoral immune response against Aβ, and the inflammatory cytokine responses (Th1 and Th2) using ELISA and QRT-PCR. To minimize the toxicity of the extracted CG, we addressed the liver toxicity in response to the CG extract treatment in Tg mice using relevant markers, such as aspartate aminotransferase (AST)/ alanine aminotransferase (ALT) measurements in serum. The EA-CG extract significantly reduced the Aβ burden, the concentration of soluble Aβ40/42 protein, and fibril formation in the hippocampus and cortex of the Tg mice treated with EA-CG (50 mg/kg BW/day) for 6 months compared with the Tg mice treated with a normal diet. Additionally, the profile of anti-inflammatory cytokines revealed that the levels of Th2 (interleukin-4 (IL-4) and interleukin-10 (IL-10)) cytokines are more significantly increased than Th1 (interferon-γ (IFN-γ), interleukin-2(IL-2)) in the sera. These results suggest that the EA-CG fraction induces IL-4/IL-10-dependent anti-inflammatory cytokines (Th2) rather than pro-inflammatory cytokines (Th1), which are driven by IL-2/IFN-γ. With regard to the immune response, EA-CG induced an immunoglobulin IgG and IgM response against the EA-CG treatment in the Tg mice. Furthermore, EA-CG significantly ameliorated the level of soluble Aβ42 and Aβ40. Similarly, we observed that the fibril formation was also decreased by EA-CG treatment in the hippocampus and cortex after quantitative analysis with Thioflavin-S staining in the Tg brain tissues. Taken together, our findings suggested that Maysin and its derivative flavonoid compounds in the EA-CG fraction might be beneficial therapeutic treatments or alternative preventative measures to adjuvant for boosting humoral and cellular include immune response and anti-inflammation which may lead to amyloid plaque accumulation in Alzheimer’s patients’ brains.

## Introduction

Alzheimer’s disease (AD) is a complicated neuronal metabolic dysfunction disease that is associated with the induction of inflammation due to microglia cell activation, a loss of synaptic receptors, and neuronal cell loss, that leads to memory loss due to brain lesions. Amyloid plaques are also present in AD, which consist of different toxic components of Aβ40 and Aβ42. These toxic components reflect genetic alterations, including the β-amyloid precursor protein (APP; Chromosome 21), the presenilin genes (PSEN1, chromosome 14; PSEN2, chromosome 1), Tau (Chromosome 17), apolipoprotein E (ApoE, Chromosome 19), and non-genetic alterations. Non-genetic alterations include environmental factors, aging, hypertension, inflammation, diabetes, malfunction of lipid metabolism, psychological stress, bacterial and virus infection and heavy-metal intoxication [1–7].

Oxidative stress is a key factor that disrupts the cellular defense machinery, which alters different types of transmembrane proteins (i.e., APP, NADPH oxidase) and increases metabolic modulators (i.e., β-amyloid, glutamate, and [Ca2+]). This alteration results in the dysfunction of synapses, autophagy, and proteasome activity. This triggers various pathological manifestations such as the formation of senile plaque, neuronal cell loss, mitochondrial dysfunction, and inflammation with the activation of microglia in AD or dementia. Increasing lines of evidence demonstrates that oxidative stress is associated with APP mutations, which result in the accumulation of amyloid β-protein (Aβ), the major component of amyloid plaques. Oxidative stress may be a causative factor that stimulates neuronal cell dysfunction in the development of AD pathogenesis [8–10]. Environmental intoxication (i.e., contamination of food, air, and water by metals or bacterial and viral infection), a non-genetic risk factor, may cause a prime catalyst for metabolic or immune disruption. The causative toxic substances, which likely initiate ROS or RNS production, disrupt the cellular defense system, such as the redox machinery and immune surveillance, in AD. Notably, it has been reported that the mortality rate due to infection has increased in elderly population suffering from Alzheimer’s disease [11].

Currently, the FDA approved few AD drugs, such as acetylcholinesterase inhibitors, N-Methyl-D-aspartate (NMDA) receptor antagonists, monoclonal antibodies for anti-Aβ, inhibitor for BACE, inhibitor for RAGE receptor and the combination drug of cromolyn sodium and ibuprofen [12]. In the past few decades, various natural compounds (phytochemicals and herbs), naturally occurring polyphenol (resveratrol, trans-3, 4', 5-trihydroxystilbene), and nutritional supplements (i.e., cinnamon extract and savory) with anti-oxidant and anti-inflammatory activities have shown potential as a beneficial counteractive approach to prevent Aβ neurotoxicity by inhibiting oligomeric formation, alternative counter measure of Tau malfunction as well as correcting memory impairment [13–16]. In previous studies, compelling reports suggested that *Centipedegrass* (CG), which originates from China and South America, contains C-glycosyl flavones and phenolic constituents as the biologically active structural skeleton. Furthermore, it was reported that Maysin (a flavone C-glycoside from corn silks and maize) and its precursor chemical components held antibiotic activity that is likely to inhibit the growth of the fall armyworm larva [17] and to resist ear damage by the corn earworm [18]. Also, it was suggested that total phenolic content of silk extracts that contain flavonoids, such as Maysin inhibited non-enzymatic glycation and the formation of advanced glycation end products (AGEs), as well as having anti-fungal effects [19]. Moreover, we explored the potential anti-Alzheimer’s effect of a CG extract, in which Maysin and its derivatives could exert protective effects for neurons by inhibiting oligomeric Aβ42 aggregation and β-Secretase (BACE) and decreasing neuronal toxicity by using neuronal PC12 cells as an in vitro model [20]. BACE, a potential drug candidate was examined its functional advantage for early phase preclinical familial AD patients along with inhibitor of RAGE (advanced glycation end products) in a Phase III clinical development stage [12]. In this study, our findings indicate that the natural Maysin and its derivative flavonoid compounds in EA-CG extract significantly prevented AD pathogenesis. It also demonstrated reduction of soluble Aβ burden in brain and mitigates formation of amyloid plaques outside the neurons by enhancing the immune response associated with Th2-skewed cytokine response in an APP/PS1 Tg Alzheimer’s disease mouse model.

## Materials and Methods

### Maysin and its flavonoid derivative compounds from *Centipedegrass* (CG)

Isolation, Purification and yield of Maysin and its derivative from *Centipedegrass* (CG) were described by Song et al. in-detail as previously [20].

### Animals

The animals were treated and maintained in accordance with the Animal Care and Use Guidelines of Gyeongsang National University, South Korea. All experimental protocols were approved through the Institutional Animal Care and Use Committee of Gyeongsang National University (approval number; GNU-130315-M0025). The B6C3-Tg (APPswe, PSEN1dE9) 85Dbo/J strain of AD mice was obtained from Jackson Laboratory (Bar Harbor, Maine, USA). All animal were housed in each cage under a 12 h light/dark cycle (light on 07:30 AM-19:30 PM) at a constant temperature (23 ± 1°C) and relative humidity (60 ± 10%) and were provided food and water ad libitum. All mice were allowed free access to their respective diets and drinking water for six months. Two groups of mice (n = 10 mice, 10 males per group, 10 week old mice) were treated for six months before being sacrificed. The mice in the first group were fed a control diet and administered PBS. The Tg mice in the second group were administered EA-CG (50 mg/kg BW) daily. We were not able to measure the food intake for individual mice because the food was provided in a common tray. Mice were food deprived overnight prior to blood collection and euthanized by ketamine (40 mg/kg) plus xylazine (2 mg/kg).

### Toxicology study using biochemical analysis

The biochemical analyses were performed using commercial kits (Asan Pharmaceutical, Co., Korea). Blood was collected 12 h after the last EA-CG administration. After collect sera from each of EA-CG treated Tg mice by centrifugation at 10,000 x g for 10 min following overnight incubation, the levels of aspartate aminotransferase (AST) and alanine aminotransferase (ALT) in the sera were detected with a DRI-CHEM analyzer (Fujifilm, Japan), respectively. The quantitative data was presented with Unit/L.

### Quantification of anti-Aβ immunoglobulins and cytokines by Enzyme-linked immunosorbent assay

Blood was collected from each group of EA-CG treated Tg and /or control Tg. After incubating blood at 4°C for 30 min, the blood was centrifuged at 10,000 g for 10 min, and then, serum was collected and stored at -80°C until it was used. The anti-Aβ immunoglobulins (IgG and IgM) in mouse serum were also measured by Enzyme-linked immunosorbent assay (ELISA), according to the manufacturer’s protocol, as previously described by Kim et al. [21]. Briefly, Microtiter immuno plate (Nunc, 8 well flat bottom; Rochester, NY) were coated with peptide Aβ 1–42 (5 μg/ml) in 50 mM carbonate buffer pH 9.6 overnight at 4°C and rinsed three times with washing buffer [phosphate-buffered saline (PBS) containing 0.05% Tween-20]. Microtiter immuno plate were treated with blocking buffer (5.0% goat serum, 1% BSA and 0.05% Tween-20 in PBS) for 1 h at room temperature (25~27°C). The serum samples were diluted with PBS and added to each wells in Microtiter immuno plate. After incubation for 2 h at room temperature with shaking gently, the plates were washed five times with the washing buffer and incubated for 1 h with an appropriate horseradish peroxidase (HRP)-conjugated detection antibody. The detection antibodies (Zymed, CA) were diluted in the blocking buffer at 1:2000 for anti-mouse IgG and IgM. After washing, plates were incubated with Chromogenic substrate (3,3′,5,5′-tetramethylbenzidine, TMB) (Pierce Biotechnology, IL USA) for 15~30 min and the reaction was stopped with the addition of 1N H2SO4. The optical densities at 450–550 nm were analyzed using a microreader (Versa max, Molecular Devices, CA) and linear regression was confirmed by 6E10, monoclonal anti-Aβ antibody, as positive internal control. It means the concentrations (μg/ml) of the serum titers presented here reflect the concentrations of 6E10 antibody. Comparison of treatment groups was performed by ANOVA and two-tailed Student’s t-test. *P < 0.05 indicates statistically significant.

### Quantification of cytokines using Enzyme-linked immunosorbent assay

T cell specific cytokines (i.e., Interferon-γ (IFN-γ), interleukin (IL)-2, IL-4, and IL-10) in mouse serum were also measured by Enzyme-linked immunosorbent assay (ELISA) using 1x TMB as a chromogenic substrate, according to the manufacturer’s protocol (eBiosciecne, San Diego, CA). Optic densities at 450 nm were determined using a microreader (Versa max, Molecular Devices, CA) within the linear regression. Each sample was measured in triplicates. The minimum detectable concentrations were 2 pg/ml for IL-2, 15.6 pg/ml for IFN-γ, 4 pg/ml for IL-4, and 32 pg/ml for IL-10.

### Quantification of soluble Aβ40 and Aβ42 by Enzyme-linked immunosorbent assay

For quantitation purposes, we used a highly specific and a sensitive sandwich enzyme-linked immunosorbent assay (ELISA) which was performed as described previously by Kim et al. [22] with some modifications First, we quantified insoluble Aβ40 and Aβ42 in the brain homogenates extracted with TBS (50 mM Tris-HCl, pH 7.6, 150 mM NaCl) or 2% SDS and 70% formic acid, respectively, as previously described [21]. A frozen hemisphere of the brain tissue was homogenized with a homogenizer in 1 ml of TBS/complete protease inhibitors (Roche, Mannheim, Germany), and then centrifuged at 100,000 g for 1 h at 4°C using an Optima TLX ultracentrifuge (Beckman Coulter Inc., CA). The TBS supernatants were stored at -80°C and the pellets were homogenized in 1 ml of 2% SDS/TBS with protease inhibitors (Roche), and then centrifuged at 100,000 g for 1 h at 25°C following 15 min incubation at 37°C. The pellets (corresponding to the insoluble fraction) were washed once, extracted further with 1 ml of 70% formic acid, and then centrifuged at 100,000 g for 1 h. The supernatants from the insoluble 70% formic acid extracts were neutralized with 1 M Tris-HCl, pH 8.0 at a dilution of 1:20. Then, the buffer soluble whole brain samples include neocortex and hippocampus was analyzed for Aβ levels using an ELISA kit (Invitrogen Corporation, Carlsbad, CA), according to the manufacturer’s instructions. The supernatant was diluted with a standard dilution buffer at 1:2000 (Aβ40) or 1:400 (Aβ42) and measured according to the manufacturer’s instructions. The obtained values were corrected for the wet weight of each brain sample and expressed as μg/mg brain.

### Detection of Aβ plaque by immunohistochemistry and Aβ fibrils burden with Thioflavin-S staining

The mice were deeply anesthetized with sodium pentobarbital and cardinally perfused with 0.9% chilled saline followed by 4% chilled formaldehyde in 0.1 M phosphate buffer (pH 7.4). The mouse brains were quickly removed and fixed in 4% paraformaldehyde for 24 h at 4°C. The brains were post-fixed overnight in 30% sucrose in 0.1M PBS. For preparing coronal mouse brain from APP/PS1 mice, brain section (35 μm thick) were cut include interested areas such as the cortex and hippocampus a freezing-stage cytochrome and kept them 0.1M PBS at 4°C. Sections were further processed for immunohistochemistry. Briefly, for detection Aβ42 and Aβ40 protein in the brain, immunohistochemistry was performed. First, elimination of endogenous peroxidase was performed by treatment of 1% H_2_O_2_/10% methanol in Tris-buffered saline (TBS) for 30 min at room temperature (RT). After washing section with 0.1M Tris-buffer (pH 7.4), sections blocked with 5% fetal bovine serum in 0.1M TBS with 0.5% triton-X 100 (TRB-T) for 1h at RT to reduce non-specific protein binding. Then, sections were incubated with primary antibody such as a mouse anti-Aβ antibody (6E10, Mouse Monoclonal antibody) (1:200; Covance, San Diego, CA) in TBS-T for overnight at 4°C. The sections were rinsed in 0.1M TBS containing with 1% serum and incubated with fluorescence conjugated second antibody for 30 min at room temperature. The secondary antibody used for fluorescence microscopy was Alexa Fluor 488-conjugated anti-mouse IgG (1:200, Invitrogen, Camarillo, CA). The section was coverslip with prolong gold anti-fade mountant (life technologies, Carlsbad, CA) and capture image under a confocal microscope. Those section were also performed counter staining with Hematoxylin and eosin (H&E) staining using brain collected from Mo/Hu APPswe PS1dE9 mice control (n = 3) and treated with EA-CG (n = 6) for safety evaluation in histological views. Four left hemisphere sections of brain including those from the cerebrum, and hippocampus were presented on each slide after two slides from each mouse were examined. Using paraffin embedded sections, first conduct de-paraffinized the section and washing them standard protocol as followings: xylene for 10 min, washing sections for 5 min in a serial dilution alcohol (from 95% to 50%), then washing sections for 10 min with double distilled water, and three times at 5 min each with phosphate-buffered saline (PBS, pH7.4). The brain sections (10 micron) were also treated with 1% Thioflavin-S (Sigma Aldrich, St Louis, MO) followed by destaining in 70% ethanol to detect the Aβ fibrils in hippocampus and cortex. Morphometric analysis using histology samples (1% Thioflavin-S stained samples) for quantification of amyloid fiber in hippocampus and cortex area, stained area was captured and then, performed quantitative analysis using Olympus BX61 automated microscope, Olympus fluoview system and the image pro plus v4 image analysis software (Media cybernetics, silver spring MD) which could capable of color segmentation and automation via programmable macros. Using coronal brain sections (n = 4), each section was separated by approximately consecutive 300 μm interval from each mouse were analyzed with each staining method at approximately forty fields (1 mm^2^ per each, using a 10X objective and a 1X eyepiece lens). Amyloid burden was expressed as a percentage of total area covered in hippocampus or neocortex by Thioflavine-S fluorescence, separately. Data were presented as mean ± standard error in a bar graph.

### Semi-quantitative RT-qPCR

The hepatic tissue or brain tissue was isolated and soaked in RNAlater^®^ Tissue Collection: RNA, Stabilization Solution (Ambion, Austin, TX) at 4°C overnight and then moved to -80°C. These tissues were homogenized in TRIzol reagent (Invitrogen, CA) to isolate the RNA. The RNA samples were treated with RNase-free DNase (Qiagen, Valencia, CA) for 15 min at room temperature, and the total RNA was purified using QIAGEN RNeasy columns. The complementary DNA (cDNA) was generated from 2 μg of the total RNA in a total volume of 20 μl using the SuperScript^®^ III First-Strand Synthesis Kit (Invitrogen) according to the manufacturer's protocol. The target cDNA was amplified using fluorescent SYBR dye and specific primers as followings: For Pro-inflammatory marker in hepatic tissue, interleukin (IL)-1α, forward 5’-AGGAGAGCCGGGTGACAGTA-3′ and reverse 5′-AACTCAGCCGTCTCTTCTTCAGA-3′; IFN-γ, forward 5’-TGAACGCTACACACTGCATCTTG-3’ and reverse 5’- GTTATTCAGACTTTCTAGGCTTTCAATG-3’; β-actin (control), forward 5'-GACAACGGCTCCGGCATGTGCAAAG-3' and reverse 5’-TTCACGGTTGGCCTTAGGGTTCAG-3’; for Pro-inflammatory marker Brain tissue, TNF-α forward 5’-TCTTCTCGAACCCCGAGTGA-3’ and reverse 5’-CCTCTGATGGCACCACCAG-3’; IL-1β forward 5’-AGGTGCTCATGTCCTCATCC-3’ and reverse 5’-CAGGCAGGCAGTATCACTCA-3’; GAPDH forward 5’-GTCATCATCTCTGCCCCCTCTGCTG-3’ and reverse 5’-CGACGCCTGCTTCACCACCTTCTTG-3’. The PCR products were also confirmed after separate follow by electrophoresis on a 1.5% agarose gel for 30 min at 100 V. The gels were stained with 1 mg/ml Ethidium bromide and visualized with UV light using the Bio-Rad Gel Doc image analysis software (Bio-Rad Laboratories Inc., CA, USA). Gene expression was normalized to β-actin as a reference gene. Quantified mRNA of each interested gene was normalized to β-actin mRNA level (ΔCt) and relative to the appropriate internal control (ΔΔCt) expressed as fold induction or decrease of control. The data were analyzed using CFX 96 real-time PCR instrument according to manufacturer’s software manual (BioRad, Hercules, CA, USA).

### Statistical analysis

The data are expressed as the means ± S.E.M. The significant differences between the EA-CG-treated and control (PBS) Tg mice were performed by Sigma Plot software (San Jose, CA, USA) using analysis of variance (ANOVA) and two-tailed Student’s t-test. A value of *p < 0.05 was considered statistically significant; n.s. mean not significant.

## Results

### CG extract (EA-CG fraction) induces an immune response in APP/PS1 transgenic (Tg) mice

**Fig 1:**
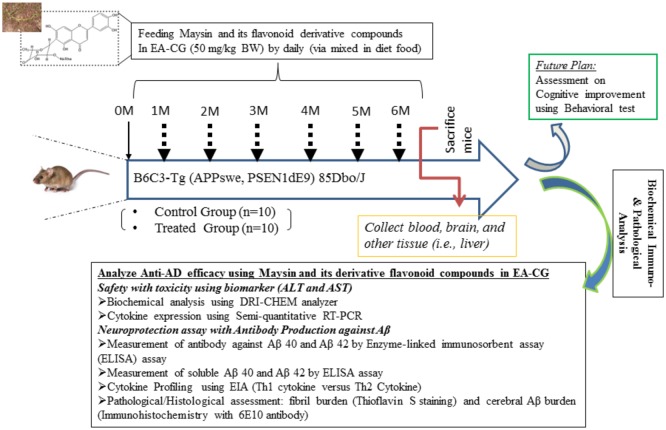
Assessment workflow of novel Anti-AD effectiveness using Maysin and its flavonoid derivative compounds on APP/PS1 AD mice model. Complication of traumatic brain injury is associated with inflammation and neural cell death from induction of Aβ 42/40 formation likely due to unfolded cerebral plaque or fibril form which results from genetic alteration in APPswe and PSEN1dE9 in the 85Dbo/J mice model for Experimental AD study. Experimental design for treatment and sampling procedure as the objectives in animal model to understand anti-AD of Maysin flavonoid with respect to prevention and/or prophylactic effect against Aβ40/42 mediated neuronal-toxicity and functional delineation as the assessment workflow in animal study are presented.

To test the efficacy of natural flavonoid compounds, we have isolated Maysin and its derivative flavonoid compounds by HPLC and gel filtration followed by elution with ethyl acetate soluble fraction (the so-called EA-CG fraction) from *Centipedegrass* (CG). In the biochemical analysis profile, it appeared that Maysin might be the major chemical component in the EA-CG fraction isolated from CG. Briefly, the isolated crude solvent extract of CG was further purified by analytic high-performance liquid column chromatography and size-dependent chromatography such as the gel filtration method. After quantification of each of the five chemical components, including Maysin and its derivative flavonoid compounds in the EA-CG fraction (for example, Luteolin, Isoorientin, Rhamnosylisoorientin, Derhamnosylmaysin, and Maysin), their production weight per 100 mg extracts was determined as the purification yield from each batch, as described in detail in a previous report [20]. Fig 1 show it has delineated a series of studies in the exploration of anti-AD lead compounds like Maysin, and its derivative flavonoid among medicine alternatives of pre-screened natural herb, CG, are designed and conducted following designated treatment protocol and sampling. This is to determine the effect of immune modulation of CG flavonoid compounds on prevention and/or prophylactic efficacy against amyloid plaque/fibril formation due to genetic factors while following relevant assessment strategies such as biochemical, immunological, patho-histological in the B6C3 Tg (APPswe and PSEN1sE9) 85 Dbo/J as an animal model for AD.

**Fig 2:**
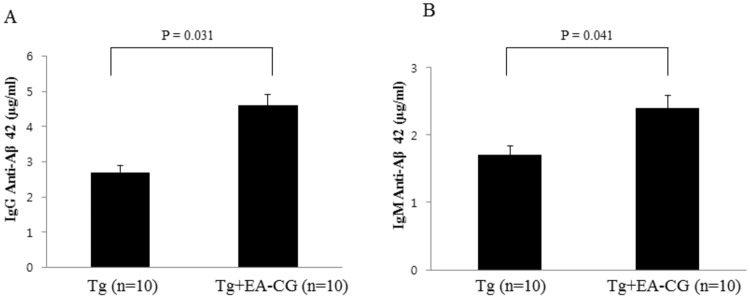
Effect of Centipedegrass (CG) extract on humoral immune response in APP/PS1 transgenic (Tg) mice. The CG ethylacetate (EA-CG) fraction contains five flavoglycosides, including Maysin and other phytochemicals, as described in a previous study [20]. Mouse sera were isolated from the EA-CG-treated and control Tg animal subjects (Mo/Hu APPswe PS1dE9) after feeding with admixture in diet food for 6 months (n = 10 mice per each group; all male and 10 weeks old mice). (A, B) To measure the beta amyloid dependent antibody response, using a sandwich ELISA, quantitation was performed utilize the Aβ1–42 synthetic peptide as the coating antigen and HRP-conjugated secondary IgG or IgM as a secondary antibody. Immunoglobulin IgG (A) and Immunoglobulin IgM (B), markers of an immune response against the EA-CG extract, were measured by ELISA, which was described in detail in the Materials and Methods. The values were presented as the means ± S.E.M. *p < 0.05 means statistically significant.

To determine whether natural flavonoid compounds such as Maysin and its precursor chemicals (labeled as EA-CG fraction) induce a humoral immune response, we have measured the IgG or IgM antibody production as a representative indicator of the immune response using an enzyme-linked immunosorbent assay (ELISA). Briefly, after dividing the mice into two groups, we fed the APP/PS1 mice (10-weeks old, all male mice) the EA-CG fraction, which contains Maysin and its precursor chemicals, in a mixture with the normal diet (50 mg/kg BW, n = 10 mice) and the control Tg mice (n = 10 mice) were fed with the normal diet for 6 months. Six months later, the sera were isolated from the mouse blood by centrifugation, and the IgG and IgM levels were determined to identify the immune response against the Aβ1–42 peptide using an HRP-conjugated secondary antibody specific for mouse IgG or IgM, respectively. As shown in Fig 2A, the immune response of the EA-CG-fed mice increased to 4.5 ± 0.35 μg/ml IgG in the sera, while the normal diet-fed mice produced 2.7 ± 0.26 μg/ml IgG (p = 0.031, IgG response, 1.67-fold induction compared to the control (PBS)). Interestingly, the immune response of IgM in the sera also increased to 2.3 ± 0.27 μg/ml in the EA-CG-fed mice compared with 1.7 ± 0.19 μg/ml in the control normal diet-fed mice (p = 0.041, IgM response, 1.35-fold induction) (Fig 2B). The difference between the control IgM and EA-CG treated IgM levels was significant (*p < 0.05 to control IgM vs EA-CG Treated IgM). These results indicate that Maysin and its precursor chemical components in the EA-CG fraction can trigger a humoral immune response by inducing an IgG antibody response. In addition, we also observed a slight induction in the IgM immune response after six months of feeding with natural flavonoid compounds utilize EA-CG extracts in the APP/PS1 Tg mice. This suggests that the induction of IgG and IgM isotypes with natural Maysin flavonoid compounds (Maysin and its derivatives in CG) may play a role in the humoral immune response using APP/PS1 Tg AD mouse model with six months’ treatment as a feeding timeline.

### Natural Maysin flavonoid compounds in the EA-CG fraction has no harmful hepatic effects

To clarify the effect of the solvent-extracted natural bioactive compounds on histopathological damage in the EA-CG-treated APP/PS1 Tg experimental animals, we first examined the hepatic toxicity using two different assays: an enzymatic assay of the tissue transaminases (ALT and AST) and a gene expression profile using RT-PCR for representative markers of pro-inflammatory cytokines (i.e., IL-1α and IFN-γ). First, we measured if EA-CG compounds may insult on hepatocellular damage in the serum levels with respect to ALT and AST transaminases in EA-CG-fed and vehicle-treated (PBS) Tg mice using an enzymatic ALT/ALS assay kit, according to the manufacturer’s instruction.

**Fig 3:**
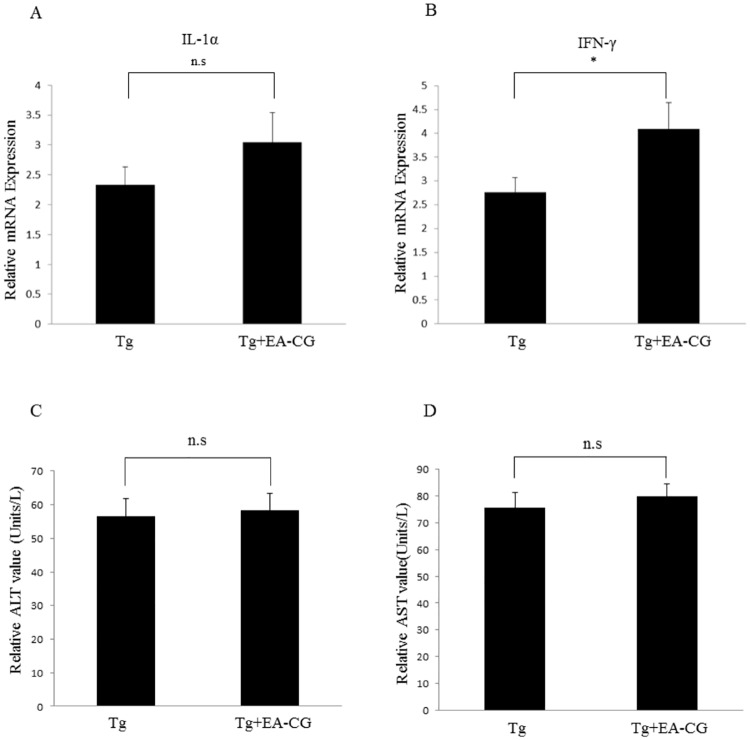
Effect of CG extracts on liver toxicity using aspartate/alanine aminotransferase activity in Tg mice. The Mo/Hu APPswe PS1dE9 mice were fed with or without EA-CG (50 mg/kg BW) for 6 months. The brain were isolated from the EA-CG treated and control (PBS) groups (n = 3 mice per each group). After the brain was divided in half, we tested the gene expression profile of the inflammatory cytokines IL-1α (A) and IFN-γ (B) as markers of the cellular response by semi-quantitative RT-PCR. The fold change was presented after normalization to the housekeeping gene β-actin). *p < 0.05; n.s. mean not significant. The levels of Aspartate aminotransferase activity (AST, Units/L) and Alanine aminotransferase activity (ALT, Units/L)) were measured in the sera by centrifugation after obtain mice blood from each group, and then the relative value (U/L) of ALT (C) or AST (D) as hepatotoxicity markers were measured and presented after comparison to the controls (PBS groups), according to the manufacturer’s instructions. The values were presented as the means ± S.E.M (n = 3 mice per each group). n.s. mean not significant.

Consequently, we also analyzed the alterations in the inflammatory response using tissue specimens from the EA-CG-treated and control (vehicle-treated) APP/PS1 Tg mice. After performing the RT-qPCR with specific primers to the amplify genes as described in materials and methods, we analyzed the mRNA level of pro-inflammatory cytokines (i.e., IL-1α and IFN-γ) using total RNA in hepatic tissue from the EA-CG-treated and control (PBS)-treated Tg mice and presented the fold change in the gene expression after normalization with the housekeeping gene. As shown in Fig 3, we observed that the level of ALT and AST in the serum was similar in the Tg mice that were fed a normal diet and the Tg mice fed with EA-CG (Fig 3C and 3D). In another experiment with relevant biomarker gene expression, we observed that the EA-CG fraction induced the expression of pro-inflammatory cytokine mRNAs, including IL-1α (2.3- vs. 3.0-fold change; control (PBS) Tg mice vs. EA-CG-fed Tg mice, *n.s; p = 0.0589) and IFN-γ (2.7- vs. 4.0-fold change; control (PBS) Tg mice vs. EA-CG-fed Tg mice, *p < 0.05). The results from the gene expression profile suggest that Maysin and other phytochemical components in the EA-CG fraction slightly triggered the pro-inflammatory cytokine IFN-γ as a skewed T cell response (Fig 3B). In contrary induction of IFN-γ mRNA expression compared to control (Tg alone), the IL-1α mRNA expression not significantly increased after normalization with the β-actin housekeeping gene (Fig 3A). Although the EA-CG fraction may cause an IFN-γ-dependent pro-inflammatory response in liver tissue, there is no significant change in IL-2 with regard to organ injury, based on the hepatic tissue (data not shown).

### Natural Maysin flavonoid compounds in the EA-CG fraction induces a Th2-skewed cytokine response

**Fig 4:**
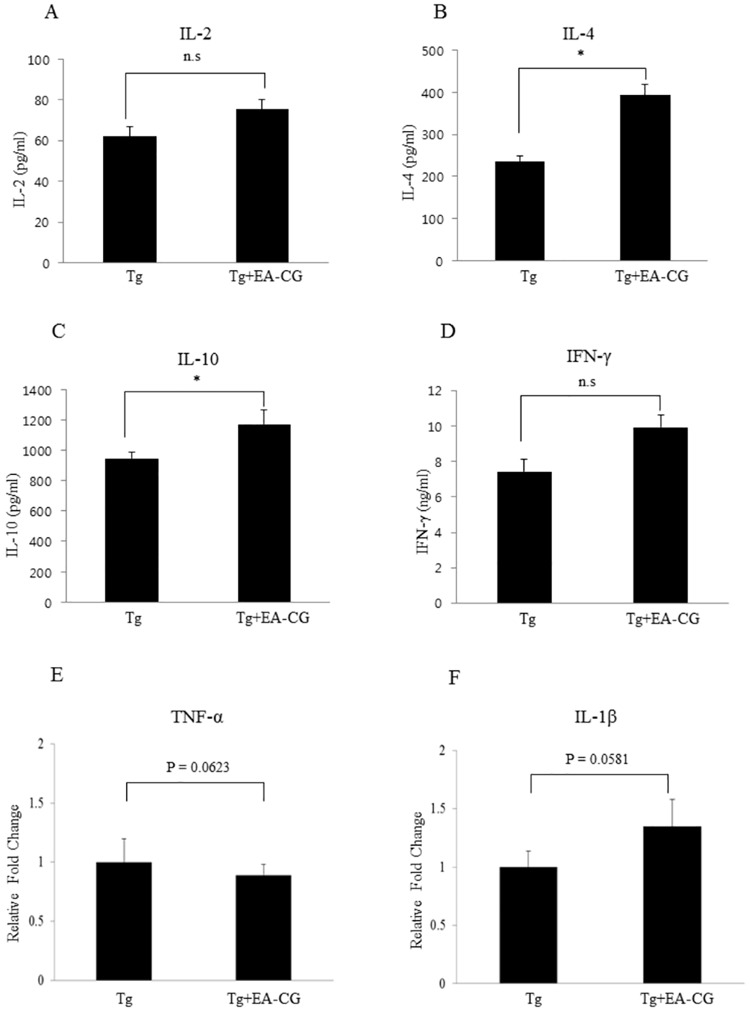
Comparison cytokine profile between Th1 and Th2 CG in the Tg brains by the CG extracts. The T cell-mediated inflammatory response, including Th1 (IL-2 and IFN-γ) (A, D) and Th2 (IL-4 and IL-10) (B, C) cytokines, was measured by a cytokine ELISA using the EA-CG-treated Tg (Mo/Hu APPswe PS1dE9) mice compared to the control (PBS) treated Tg mice. After the mice sera were isolated by centrifugation, the levels of the pro-inflammatory cytokines (Th1: IL-2 and IFN-γ) and anti-inflammatory cytokines (Th2: IL-4 and IL-10) were quantitated and analyzed the difference sera between in the EA-CG-treated and vehicle (PBS)-treated control Tg (Mo/Hu APPswe PS1dE9) mice. For quantifying pro-inflammation difference between control Tg and Tg+EA-CG in gene expression profile, gene of interest (TNF-α (E) and IL-1β(F)) was presented as relative fold change after normalized with housekeeping gene (GAPDH). The values were presented as the means ± S.E.M (n = 10 mice per each group). *p < 0.05. Tg vs. Tg+EA-CG; n.s. mean not significant.

To determine whether the EA-CG fraction from CG may induce T-cell activation, most likely T lymphocyte helper (Th) 1 or Th2 cytokine responses, we quantified the secreted Th1 (pro-inflammatory) or Th2 (Anti-inflammatory) profiles using a cytokine ELISA assay and sera from the EA-CG-treated and control Tg mice. The cytokine response was shown in Fig 4. The Th2 cytokine profile (Fig 4B and 4C) included the IL-4 serum levels (220 ± 21.0 vs. 390 ± 40.0 pg/ml; control (PBS) Tg mice vs. EA-CG-treated Tg mice) (P = 0.045) and IL-10 serum levels (950 ± 80.4 vs. 1150 ± 110.6 pg/ml; control (PBS) Tg mice vs. EA-CG-treated Tg mice) (P = 0.036); the Th1 cytokines (Fig 4A and 4D) included the IL-2 serum levels (62 ± 2.5 vs. 70 ± 4.0 pg/ml; control (PBS) Tg mice vs. EA-CG-treated Tg mice) (P = 0.0568) and IFN-γ serum levels (7.2 ± 0.8 vs. 10 ± 0.9 pg/ml; control (PBS) Tg mice vs. EA-CG-treated Tg mice) (P = 0.061). Based on the quantifying the Th1 and Th2 cytokines levels, we hypothesized that the EA-CG extract may exert its efficacy in preventing AD by promoting significantly the Th2 cytokine response rather than the Th1 response in APP/PS1 Tg mice after six months of feeding with Maysin and its derivatives.

To define further analysis with regard to inflammation and neuroprotection aspects, we measured the difference mRNA cytokines expression using relevant gene markers (i.e., TNF-α and IL-1β) between control Tg mice and EA-CG-treated Tg mice using RT-qPCR. There is no significant difference in gene expression profile represented by fold change after normalized with GAPDH as followings: for example, expressions of TNF-α mRNA, control Tg mice and EA-CG-treated Tg mice (1.0 ± 0.2 vs. 0.89 ± 0.09, P = 0.0623) and for IL-1β, control Tg mice and EA-CG-treated Tg mice (1.0 ± 0.14 vs. 1.35 ± 0.23, P = 0.0581). Taken together, these result indicated that the EA-CG extract containing Maysin and its derivative flavonoid compounds could exert in preventing or ameliorating amyloid aggregation by enhancing Th2 cytokine production without coincidence event likely to activate pro-inflammatory T cell signaling.

### Amyloid plaques are reduced in Maysin flavonoid compounds treated APP/PS1 Tg mouse brains

**Fig 5:**
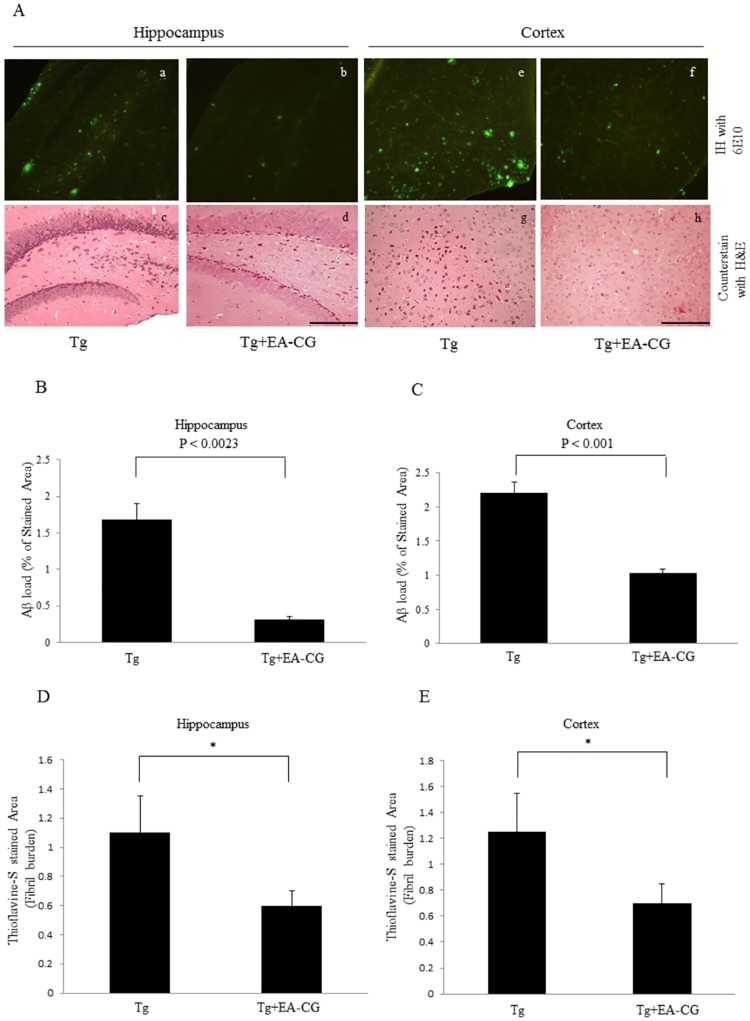
Comparison of the amyloid burden in the Tg brains by the CG extracts. (A) The amyloid plaques were identified by immunohistochemistry (IH) and H&E counterstaining. The CG extract-treated (EA-CG fraction) and control Tg brain sections were formalin fixed and subjected to immunohistochemistry with the 6E10 antibody that interacts with the Aβ1–16 epitope (monoclonal antibody) in the hippocampus (a, control; b, EA-CG treated) and cortex (e, control; f, EA-CG-treated). Concomitant with the IH staining, other hippocampal (n = 6) (c, vehicle; d, EA-CG-treated) and cortical (n = 6) (g, vehicle; h, EA-CG-treated) sections from the Mo/Hu APPswe PS1dE9 mice were subjected to H&E counterstaining. The scale bar indicates 200 μm (a through h). The amyloid load (% of stained area) as a quantitation result was decreased by the CG extracts (B. hippocampus area; C. cortex area). The area in the hippocampus (D, vehicle alone vs. EA-CG) and cortex (E, vehicle alone vs. EA-CG) was quantified by comparing the morphometric analysis of the Thioflavin-S-stained area between the vehicle-treated vs. EA-CG-treated Mo/Hu APPswe PS1dE9 brain sections. The values (%) shown were the means ± S.E.M. *p < 0.05, Tg vs. Tg+EA-CG.

The amyloid plaque burden is a key pathological phenomenon in Alzheimer’s disease that accelerates neuronal damage and impairs cognition. We have evaluated the differences in the amyloid plaque burden include diffuse and fibrillary amyloid deposit in the brains of the EA-CG-treated and control Tg mice by using immunofluorescence staining with the specific antibody 6E10. Antibody 6E10 is a monoclonal antibody that was generated against the Aβ1–16 epitope. Representative image was shown in Fig 5A, we also observed mitigation of amyloid burden in the APP/PS1 Tg mice in both the hippocampus (Average of percentage of Area as Aβ load: 1.704 ± 0.209 vs. 0.287± 0.081 in Tg vs Tg+EA-CG, P < 0.0023) (Fig 5B) and cortex (Average of percentage of Area as Aβ load: 2.189 ± 0.189 vs. 1.025 ± 0.068 in Tg vs Tg+EA-CG, P < 0.001) (Fig 5C) was significantly reduced in the EA-CG-treated (50 mg/Kg BW) Tg mice group compared to the control Tg mice that were fed a normal diet. To monitor the patho-histological relevance, each brain section, including the hippocampus and cortex, was counterstained with Hematoxylin and Eosin (H&E). These histological results indicated that EA-CG prevented amyloid plaques include diffuse and fibrillar Aβ deposits in both the hippocampus and cortex in the APP/PS1 Tg mice.

### Amyloid fibril burden are decreased by Maysin flavonoid compounds treated APP/PS1 Tg mice

To further determine whether the EA-CG fraction reduced amyloid fibril formation, we examined the brain morphology by staining utilize Thioflavin-S, which specifically binds the amyloid fibrils at hippocampal and cortical lesions. By comparing the EA-CG-treated or vehicle (PBS)-treated Tg mice, we observed that the EA-CG fraction reduced the amyloid fibril burden by 47.27% in the hippocampus (1.1 ± 0.23 in the control (PBS)-treated Tg mice vs. 0.58 ± 0.08 in the EA-CG-treated Tg mice, *P < 0.021) and 44.0% in the cortex (1.25 ± 0.31 in the control (PBS)-treated Tg mice vs. 0.7 ± 0.09 in the EA-CG-treated Tg mice, *P < 0.036). The quantitative results of the amyloid fibrils from representative images were shown in Fig 5D (hippocampus) and 5E (cortex), respectively. These results suggest that Maysin and its derivatives flavonoid compounds in the EA-CG fraction significantly prevented amyloid plaques as well as fibril formation, the key features of AD, in both the hippocampus and cortex of the APP/PS1 Tg Alzheimer’ disease mouse model.

### The cerebral Aβ load is reduced by Maysin and its derivative flavonoid compounds

**Fig 6:**
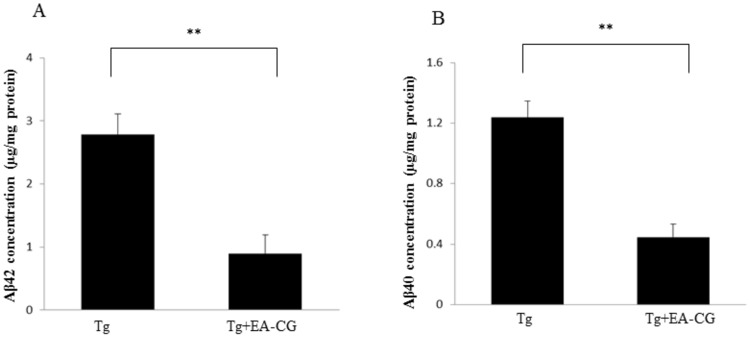
Comparison of Soluble Aβ42/40 by the CG extracts on Tg mice brain. The cerebral Aβ42(A) and Aβ40 (B) load was quantitated by capturing antigen such as Amyloid beta 42 and/or Amyloid beta 40 mediated Enzyme-Linked Immunosorbent Assay (ELISA) kit (Invitrogen, CA) using brain lysates (i.e., total brain lysate of hippocampus and neocortex area) from the EA-CG-treated or vehicle (PBS)-treated Tg (Mo/Hu APPswe PS1dE9) mice. The cerebral Aβ42 concentrations in the EA-CG-treated mice were significantly reduced compared to the vehicle-treated controls (**P < 0.01). The values shown were the means ± S.E.M. (n = 10 per group).

As shown in previous studies, the Aβ load is associated with neurotic plaques in the cerebral cortex. To determine the effect of Maysin and its derivative flavonoid compounds on the Aβ load, the total amounts of 42 and Aβ40 in the hippocampus and cortex areas on Tg brains were analyzed using Aβ42 or Aβ40 ELISA kits. As shown in Fig 6, the average Aβ42 concentration in the EA-CG-treated Tg mice was 0.80 ± 0.1 μg/mg protein. Meanwhile, the average Aβ42 concentration in the control Tg mice treated with a normal diet was 2.71 ± 0.26 μg/mg (**P < 0.0012) (Fig 6A). Similarly, the average Aβ40 concentration in EA-CG-treated Tg mice was 0.41 ± 0.09 μg/mg protein compared to control Tg mice treated with a normal diet (1.26 ± 0.13 μg/mg protein) (**P < 0.0016) (Fig 6B). These results suggested that the burden of cerebral amyloid load (i.e., hippocampus and neocortex) could be altered in the Tg mice following treatment with Maysin and its derivative flavonoid compounds, based on the reduction in the Aβ42 and Aβ40 levels in the APP/PS1 Tg brains after 6 months of treatment.

## Discussion

Alzheimer disease (AD) is a complex and common progressive neurodegenerative disease that reflects multifactorial gene mutation (i.e., APP, Presenilin (PSEN) 1 and PSEN 2, ApoE4) as a hallmark of the etiology of familial AD (FAD) and Sporadic AD (SAD) in the elderly population [23–25]. While a substantial proportion of the vulnerability for APP in AD may be accounted for by genetic factors, environmental factors include oxidative stress, mitochondrial dysfunction, and inflammation probably play a significant role [26–30]. It was also projected the genetic cause of Alzheimer disease (AD) is understood for less than 1% gene mutations (APP, PS1, PS2) in early onset FAD or 20% (APOE4 allele) in late onset FAD cases, respectively [31]. For the remainder of AD sufferers, multiple genetic and environmental factors probably play a role in contributing to disease onset, severity and progression due to promotion of amyloid-beta peptide (Aβ42/40) generation following proceeding of APP cleavage due to secretase activation [32–34]. To date, there is no cure for AD and therapeutic measures are being actively explored through small molecule likely inhibitors to three isoform of secretases and/or neutralization of amyloid-beta peptide (Aβ42/40) toxicity using biologicals, small molecules, and immunotherapy in the pharmacological active natural compounds.

It was reported the potentiality as therapeutic regimen in which anti-AD-like effectiveness of the natural flavonoid compounds in CG extract (EA-CG) in a Mo/Hu APPswe PS1dE9 Tg AD mouse model. The natural flavonoid compounds, named the EA-CG extract, which contained five phytochemicals that are the major constituents of Maysin (i.e., Luteolin, Isoorientin, Rhamnosylisoorientin, Derhamnosylmaysin, and Maysin) in which those are may represents a potential anti-BACE1 phytochemicals as non-pharmacological alternatives or diet regimen that could prevent AD-like pathological symptoms by reducing soluble β-amyloid production and inhibiting amyloid plaque formation [20]. Compelling evidence support the premise likely effectiveness of natural flavonoids as Anti-AD in which one of flavonoid compound, Luteolin (include c-glycosylated derivatives) or Isoorientin, have shown its efficacy to prevent anti-Diabetic, anti-diabetic using diet induce cognitive deficit in obesity C57Bl/6 mice [35, 20] Liu et al. reported that Luteolin exerted anti-AD effect such as impairment of spatial learning and memory capabilities, dysfunction of microvascular function, the intervene regional cerebral blood flow, result from Aβ (25–35) derived production of reactive oxygen species (ROS) using i.c.v. injection of aggregated Aβ (25–35) peptide in Male Kunming mice [36]. They explicated therapeutic efficacy of Luteolin as Anti-AD rely on the activation of cholinergic neuronal network with boosting brain-derived neurotrophic factor (BDNF) through its receptor tyrosine kinase B expression located in cerebral cortex [36]. Similarly, the impact of Luteolin on neuroprotection led by Cheng et al. was demonstrated that flavonoid compound likely Luteolin prevent signaling intervention reflects Aβ (25–35) induced neuronal cell death result from alteration of signaling molecules pertaining to oxidative stress such as ERK-p, JNK, JNK-p, P38-p and caspase 3 activations using rat primary cortical cultures [37].

Alzheimer’s disease (AD) is also known as featured pathology likely amyloid aggregates that result from oxidative stress and inflammation due to multifactorial oxidative reactions, including protein oxidation, lipid peroxidation, and ROS generation. These reactions potentially exacerbate neuro-inflammation and neuronal cell damage and may lead to neurodegeneration [38].

Similar to previous reports, it was indicated the molecular target causes multifactorial brain dysfunction as a consequence of oxidative damage, resulting in inflammation, calcium homeostasis disturbance, and an imbalance of the redox system in AD. Oxidative stress is profoundly associated with several metabolic disruptions in AD patients, such as mitochondrial dysfunction and altered activity of mitochondrial cytochrome oxidase, pyruvate dehydrogenase, and α-ketodehydrogenase [39]. Other metabolic disruptions could be associated with oxidative stress in which synaptic dysfunction induced by Aβ is internalized within membrane receptors, such as NMDA, advanced glycation of end-product receptors and/or alpha 7 nicotinic acetylcholine receptors [40].

The accumulation of toxic, soluble Aβ40/42 in the brain sequentially induces oxidative stress; for example, the early phase response to cleave Aβ includes activating mutations in APP, PS1, PS2 genes and activating β- and γ-secretases, and the late phase response to activate the secretases or decrease Aβ clearance includes oxidative stress-related factors (radicals) as risk factors (i.e., ApoE4, sortilin related receptor 1, clusterin, and complement component) [41]. It was also reported that the antioxidant and anti-inflammatory drugs GVT (Graft Versus Tumor) or MSL (monosodium luminol) suppress ts1-induced oxidative stress by up-regulating the nuclear transcription factor NF-E2-related factor 2 (Nrf2) using a murine retrovirus ts1 mutant as a model for human immunodeficiency virus (HIV)-associated dementia [42]. Interestingly, an increasing body of evidence supports the notion that Aβ oligomer species induce cognitive dysfunction that is linked to increased neurogenesis defects, synaptic damage and inflammation [43]. Moreover, mutations in the APP, PS1 and PS2 genes cause an altered protein folding process with malfunctions in the endoplasmic reticulum (ER), altered mitochondrial function and a decrease in myelin formation associated with cognitive impairments during the aging process [44].

A natural herbal is viewed as potential alternative countermeasure as AD intervention. Recent studies include our data indicate that a natural herbal medicines exert in anti-inflammatory and anti-AD-like effects in Tg AD mice, and in alleviating the pathological features that are similar to AD patients. For example, phytochemical compounds with potential anti-AD-like effects, such as Fulvic acid, prevent the formation of tau filaments [45]. Phenolic compounds (i.e., curcumin, ferulic acid, myricetin, nordihydroguaiaretic acid (NDGA), and rosmarinic acid (RA), and theaflavins (TF3), the main polyphenolic components found in fermented black tea, silibinin (silybin), a flavonoid derived from the herb milk thistle (Silybum marianum)) prevent amyloid plaques, oligomeric fibril formation and affect soluble Aβ species [46–48] in vitro and in AD model transgenic mice (Tg2576). Moreover, it was shown that natural compounds, including flavonoids or nutrient supplements contribute to mitigating the chronic cerebral inflammatory response, which may induce synaptic loss, microglia activation, and neuronal dysfunction. This is accelerated by the induction of oxidative damage resulting from the production of misfolded β-amyloid peptide and the formation of the pathological plaque and neurofibrillary tangles (NFTs) [49–50].

Longpré et al. [51] reported that the flavonoid fraction could inhibit Aβ-mediated neurotoxicity and Aβ fibril formation, which may be due to the activation of the redox-sensitive transcription factor NF-KB and the MAPK signaling pathways as a potential mode of action. In addition, two polyphenol components of pomegranate extract (punicalagin and ellagic acid) ameliorated the cognitive impairment, which may counteract the microglia activation and accelerated inflammation associated with the Aβ-induced pro-inflammatory cytokine, TNF-α, in (APP/PS1) transgenic AD mice [52]. In accord with these data, one of the CG extract compounds, Maysin has shown insecticidal activity against corn earworm [53], anti-inflammatory effects by inducting the pro-inflammatory cytokine, TNF-α, and inducible nitric-oxide synthase (iNOS) gene expression coupled with the phosphorylation of Akt and MAPK signaling in activated macrophage [54]. Similar to these scientific findings, our results also indicate that the CG compound could induce the Th2 cytokine response as a result of the enhanced levels of the anti-inflammatory profile, as shown by IL-4 and IL-10- induction in the EA-CG (Maysin and its flavonoid derivatives)-treated Tg AD mice after six months of an EA-CG diet. Furthermore, the brains from the Tg mice were showed a significant reduction in the amyloid burden, which may be affected by an enhanced anti-inflammatory cytokine response (Th-2-skewed cytokine response, but partial overlap with the Th1 cellular response) and anti-oxidant effects to reduce the generation of Aβ42/40 species as well as the humoral immune response (immunoglobulin IgG response).

Natural flavonoids include CG compound may be bi-functional intervention such as anti-oxidant and anti-inflammation as an alternative of AD therapeutic tool. However, there are some existing hurdles to designing better drugs that can answer the question as to whether intake stability and the persistence of anti-oxidant formulations along with supplements, such as vitamin E, vitamin C, and beta carotene, may reduce the risk of AD, or whether there is a need for both anti-oxidant and anti-inflammatory properties to prevent the oxidative stress-mediated aggregation of Aβ in the AD brain. Moreover, there are some discrepancies in the AD genetic factors that cause Aβ aggregation (amyloid plaques) or toxic Aβ unfolded molecules in AD patients. Interestingly, it was demonstrated that multiple polyphenol compounds are beneficial for enhancing cognitive abilities and synergistically mitigating Aβ-mediated neuropathology by reducing the total amyloid content and improving the oligomeric Aβ (oAβ)-induced long term potentiation (LTP) deficits in hippocampal slices after treatment with a combination of three polyphenolic preparations (i.e., grape seed extract, resveratrol, and Concord grape juice extract) in J20 AD mice [55].

Limitation of current study due to low yield of byproducts separation in EA-CG does not validate the difference mode of action of between Maysin (and its flavonoid compounds) and Luteolin to better understand their beneficial anti-oxidant and anti-inflammatory effects to BACE as well as cellular immune response focused on T cell biology. It may be accompanied with the reduced amyloid burden and improved cognitive functions, using behavioral experiments and the recovery of synaptic functions and related brain derived growth factor (BDNF) include its receptors in cholinergic neurons. We have not yet determined how this dual anti-AD like functions are associated with prevention of Aβ accumulation or mitigate beta amyloid plaque in brain due to the low yield of each fraction of chemicals in EA-CG extracts (i.e., Maysin and its flavonoid derivatives). There are some restrictions to reveal the real aspects of Maysin’s effectiveness with regard to the inhibition of amyloid plaques in the Tg brains as well as the amelioration of cognitive impairments using a battery of behavioral experiments in the future.

In conclusion, we first demonstrated that natural flavoglycoside compounds, such as Maysin and its precursor phytochemicals that are isolated from CG, profoundly reduce the amyloid plaque and fibril burden in the hippocampus and cortex using APP/PS1-overexpressing Tg AD mice. Strength of this study was highlighted such as the beneficiary effect of the EA-CG fraction (Maysin and its precursor chemicals) such as decrease the soluble Aβ40/42 protein concentrations in brain lysates, which may be associated with the induction of a Th2-skewed immune response by producing an immunoglobulin IgG response. Potential activity of the EA-CG fraction could significantly enhanced the Th2 (anti-inflammatory) cytokine response compared with Th1 (pro-inflammatory) cytokine response in the serum. With tissue safety issues during the six months of EA-CG treatment by feeding a mixed diet, there were no hazard symptoms appeared in the hepatic toxicity assay using Tg mice. When natural flavonoid compounds in EA-CG have proceed their cellular immune response following EA-CG treatment in APP/PS1 Tg mice, we observed that an induction of IFN-γ rather than IL-1α in the spleen and other organs (data not shown) from the APP/PS1 Tg mice occurs. However, histology data did not shown any inflammatory outcomes in APP/PS1 Tg mice fed with these flavonoid chemicals for four months. Overall, our results are the first to indicate that the natural flavonoid compounds in EA-CG extract (i.e., Maysin and its flavonoid derivatives) is a promising phytochemical therapeutic agents or prophylactic measure, which could prevent amyloid plaque/Aβ aggregation by enhancing Th2-skewed cytokines rather than Th1 cytokines, particularly IL-4 and IL-10. This may provide new insights on the role of multiple flavonoids in effective neuroprotection by enhancing IL-4 and/or IL-10 expression, which would be a molecular bridge between the anti-oxidant and anti-inflammatory activities. By providing potential evidences with respect to mitigate amyloid burden and Aβ burden in cerebral and hippocampus on APP/PS1 tg mice model, it would be permissible to consider Maysin and its flavonoid compounds in EA-CG as Anti-AD countermeasure. The affect potentially reverse the cognitive impairments contingent with prevention of BACE and oxidative stress mediated neuroinflammation by humoral immune response in AD in the future. Further experiments are required to expedite underlying mechanism in detail by which novel anti-AD, Maysin and its flavonoid derivatives compounds, counteract cerebral Aβ burden and enhance humeral and cellular immune network about production of anti-Aβ and anti-inflammatory cytokines expression and be needed to determine if mitigate cognitive impairment, too.
